# Isolation of bovine milk–derived extracellular vesicles via a capillary-channeled polymer (C-CP) fiber stationary phase

**DOI:** 10.1007/s00216-025-05824-0

**Published:** 2025-03-13

**Authors:** Carolina Mata, Jerisa M. Pimentel, Kun Huang, Alexis Stamatikos, R. Kenneth Marcus

**Affiliations:** 1 Department of Chemistry, Biosystems Research Complex, Clemson University, 105 Collings St., Clemson, SC 29634-0973 USA; 2 Department of Chemistry, Gettysburg College, Gettysburg, PA 17325 USA; 3 Department of Food, Nutrition, and Packaging Sciences, Clemson University, Clemson, SC 29634-0316 USA

**Keywords:** Bovine milk–derived extracellular vesicles (MDEVs), Capillary-channeled polymer (C-CP) fibers, Liquid chromatography (HPLC), Exosomes, Gene therapy

## Abstract

Extracellular vesicles (EVs) are released by all cell types into the extracellular environment. A subset of EVs, known as exosomes, range in size from 30 to 200 nm and are of biochemical interest due to their function as vehicles of intercellular communication. Their ability to transport proteinaceous species and genetic material at the cellular level makes them prime candidates as vectors in gene therapies. Focusing on biotherapeutics, bovine milk–derived extracellular vesicles (MDEVs) hold particular promise as an alternative to other exosome sources for therapeutics delivery. Bovine milk poses unique challenges due to the complex colloidal matrix, composed predominantly of fats and proteins like casein, which form micelles that exhibit exosome-like characteristics, specifically size and density. When faced with complex matrices like milk, conventional size/density-based isolation methods including ultracentrifugation and size exclusion chromatography struggle to provide high purity yields on practical time and cost scales. When paired with a stepwise hydrophobic interaction chromatography (HIC) gradient, polyester (PET) capillary-channeled polymer (C-CP) fibers in column and spin-down tips formats have been used effectively to isolate exosomes from highly diverse sources. Here, PET C-CP fiber columns are demonstrated to isolate MDEVs from pre-treated raw milk, yielding concentrations of 1.5 × 10^10^ particles mL⁻^1^ with purities of ~2 × 10^10^ EVs µg^−1^ protein in less than 20 min. The efficacy of the isolation process is verified by a suite of characterization methods. Implementing PET C-CP fiber columns for MDEV isolation addresses the challenges associated with conventional isolation methods, holding promise for scale-up towards therapeutic applications.

## Introduction

Exosomes, known to be excreted by all cell types, are a subset of extracellular vesicles (EVs) which range in size from 30 to 200 nm [1]. Their source diversity spans plants, animals, and bacteria, and thus has drawn the attention of researchers across the breadth of fundamental biology and biochemistry relative to cell-to-cell communication and disease propagation. EVs are released from host cells when multivesicular bodies (MVBs) fuse with cell plasma membranes and are excreted into the extracellular space where they regulate intercellular transport and communication. This process results in exosomes decorated with a protein-rich outer membrane that is reflective of their host cell of origin. It is this characteristic that draws the interest of clinicians seeking to use exosomes as biomarkers for pathological and diagnostics applications [2]. A roadblock to a better understanding of their fundamental biochemistry, utilization in medical diagnostics, and harnessing exosomes as effective vectors is their isolation from complex matrices ranging from urine, saliva, plasma, placenta material, plant material, and cell cultures. Here we address the harvesting of exosomes from bovine milk [3, 4].

The selection of the most appropriate exosome isolation method is often specific to the source of origin and likely tailored to the intended downstream application; fundamental biochemistry, clinical diagnostics, or therapeutics. Methods of EV isolation most often focus on separation based on size and density, such as the most commonly utilized ultracentrifugation (UC), where samples are centrifuged at high speeds (~ 100,000 × g) for extended periods of time (2–24 h) [5, 6]. While widely accepted, this isolation method can be wasteful and requires relatively large (multiple mL) minimum sample input volumes, often yielding low exosome returns that are impacted by co-segregation of protein aggregates, lipoproteins, and other microvesicle impurities [5]. The shear forces applied to vesicles via ultracentrifugation often results in ruptured and structurally compromised exosomes which are not viable for most downstream applications. Size exclusion chromatography (SEC) has been increasingly implemented for EV isolation, and while exosomes with fewer protein contaminants are recovered, purity remains a challenge due to lipoprotein co-elution [7]. In addition, overall low recoveries are realized due to on-column holdup of the vesicular species within the porous stationary phase beads.

Tangential (field) flow filtration (TFF), another size-based isolation method, has become a popular separation technique to isolate exosomes on a larger scale [8, 9]. This method sees less protein content and improved batch-to-batch reproducibility in comparison to other isolation methods. While TFF yields vesicles that experience less membrane shearing than ultracentrifugation, due to the nature of this separation method, exosome extracts have increased dilution factors than the other commonly implemented isolation techniques [8, 9]. The single-use/disposable nature of TFF membranes contributes to high costs, and difficulties exist in processing small sample volumes as relevant in fundamental/clinical studies. As the fields of exosome study and application expand, and the isolation of vesicles from complex matrices becomes commonplace, inexpensive and reliable methods of isolation that can handle a variety of matrices, while having low operational costs along with higher throughput and exosome purities, will be indispensable.

Bovine milk–derived extracellular vesicles (MDEVs) are touted as a near-ideal vector for therapeutics due to the ready availability of the source material, low cost, high biocompatibility, lack of immunogenicity, and resistance to denaturation in harsh environments, including those with fluctuations in temperature and pH [3, 8, 10]. Challenges associated with the isolation of EVs from bovine milk include relatively high sample viscosity and copious amounts of matrix-associated species, including somatic cells and excess amounts of fats and proteins [11]. Casein proteins make up ~ 80% of all protein content in raw bovine milk, where they form a micellar colloidal system to help facilitate the transport of important nutrients from mother to calf for growth and development [11, 12]. While effective in the transport of nutrients, casein micelles have a tendency to mirror EV properties including their size range, spherical shape, and ability to readily interact with other hydrophobic species, therefore interfering with the facile isolation of these EVs, pointing to the need for a more tailored extraction process [11, 12].

Difficulties in collapsing the colloidal system stem from calcium phosphate, which forms a core for the micelles where highly phosphorylated casein can interact and form aggregates of varying sizes [12]. The micelles are stabilized through hydrophobic and electrostatic interactions but can be destabilized through various procedures including heating to 100 °C, adding an excess of salt, and removal via differential ultracentrifugation [12, 13]. TFF has risen in popularity for the separation of MDEVs due to its capacity to produce high yields of biologically active vesicles with minimal shearing effects [3, 14]. While successful in isolating particles in high yields, this method often does not address the challenge that casein micelles pose to high-purity isolations. Specifically, when TFF is utilized for the isolation of bovine milk–derived exosomes, western blots show co-isolation of casein in the resulting exosome fractions [3].

While the above-mentioned methods are effective, they also have the potential to negatively affect exosome morphology. Destabilization of casein colloidal systems through gradual acidification, causing changes in electrostatic interactions and leading to aggregation, has proven to be effective in the disruption and precipitation of micelles, while tending to preserve the structural integrity of EVs [12, 15]. Work by Rahman et al. has shown success in precipitating casein micelles by acidification prior to the isolation of EVs by ultracentrifugation. However, EVs with rough surfaces and deterioration regarding the retention of the vesicle-bound tetraspanin proteins CD9 and CD81 were reported [16]. Husnaeni et al. studied optimal conditions for the isolation of casein from bovine milk via precipitation and filtration with a focus on obtaining casein with low water content and minimally dissolved protein for downstream nutritional applications [17].

In general, current methods of MDEV isolation require hours of separation, large volumes of samples, expensive consumables, and excess amounts of acid that can degrade EVs. As such, there are many opportunities for improvement. Previous work by Marcus and co-workers utilizing hydrophobic interaction chromatography (HIC) performed on polyester (PET) capillary-channeled polymer (C-CP) fiber columns has shown great success in the isolation of high-purity exosomes from a multitude of matrices [18]. Matrices including human urine, seminal fluid, breast milk, saliva, blood plasma, cell culture media, and plant-derived supernatants have all posed unique challenges in the isolation of exosomes due to matrix contents and varied viscosities. PET C-CP fiber columns have handled each of these matrices with ease, yielding morphologically intact, high-purity, biologically active EVs [18]. Using a 3-part, stepwise HIC gradient the elution of high-purity exosomes is achieved in less than 15 min [19]. In this protocol, the sample is injected under high ionic strength conditions (2 M (NH_4_)_2_SO_4_) wherein small molecules, salts, and sugars pass through the column unretained, and the more hydrophobic proteinaceous materials and exosomes are retained on the fiber surfaces. A second, lower ionic strength/mild organic solvent step elutes proteins, and a third mild organic solvent step elutes the exosomes. A considerable advantage of implementing PET C-CP fiber columns is their low cost and their utility. While a traditional commercial liquid chromatography column costs hundreds to thousands of dollars, PET C-CP fiber column materials cost less than $5 in total, are assembled in house, and are easily regenerated [20]. By these methods, sample volumes of 10–100 µL are easily processed in < 15 min, with recoveries of up to 10^11^ EVs on microbore scale columns. Product purities of > 10^11^ exosomes per microgram of residual protein are very common, where the accepted standard for designating an exosome isolate as “pure” is suggested to be 3 × 10^10^ vesicles per microgram of protein [21]. Current efforts are working towards column scale-up, with the goal of purifying upwards of 10^14^ exosomes at a time.

Described here is the extension of the HIC on PET C-CP fiber columns specifically to affect the isolation of MDEVs from raw bovine milk. As described above for other MDEV approaches, issues regarding the alleviation of matrix proteins, fats, and micelles are key aspects in the method development. The effectiveness of the approach is assessed using conventional EV test methods, including electron microscopy, light scattering, immunofluorescence assays, and Bradford and western blot assays. Ultimately, exosomes having an average diameter of 68 nm are derived at densities of up to 1.5 × 10^10^ particles mL^−1^, several orders of magnitude higher than particle counts reported from other MDEV isolation methods [22]. Final isolates are free from > 99% of proteinaceous matrix species, with purity values in line with the standard threshold. Overall, the method holds promise across multiple size scales, from fundamental biochemistry and process analytics as demonstrated here, extending in the future to the preparative scale.

## Materials and methods

### Chemicals and reagents

Deionized water was obtained from an Elga PURELAB flex water purification system (18.2 MΩ cm^−1^) (Veolia Water Technologies, High Wycombe, England). Laboratory-skimmed milk was syringe filtered using 0.22-µm polyether sulfone (PES) filters (FroggaBio, Toronto, Canada). Ultrapure ammonium sulfate (AMS), HPLC grade acetonitrile (ACN), and dimethyl sulfoxide (DMSO) were purchased from VWR (Sokon, OH, USA). Phosphate-buffered saline (PBS, pH = 7.4), Pierce™ Coomassie Plus Bradford assay reagent, and glacial acetic acid were purchased from Thermo Fisher Scientific (Waltham, MA, USA). β-Casein from bovine milk was purchased from Millipore Sigma (Burlington, MA, USA). To calibrate the NanoFCM, 0.25 µm fluorescent silica microspheres (QC Beads) with a particle count of 2.17 × 10^10^ particles mL^−1^ and silica nanosphere cocktails (S23M-sEV) demonstrating 4 populations at 53, 73, 96, and 120 nm were purchased from NanoFCM, Inc. (Nottingham, Nottinghamshire, UK). MEMGlow™ fluorogenic membrane probe (640 nm) dye and FITC CD81 ExoBrite flow antibodies used for immunoconfirmation were purchased from Cytoskeleton, Inc. (Denver, CO, USA) and Biotium (San Fransisco, CA, USA) respectively. All solvents used for NanoFCM were filtered using a 0.22 µm polytetrafluoroethylene (PTFE) hydrophilic syringe filter purchased from Thermo Fisher Scientific (Waltham, MA, USA). For EV quantification, a lyophilized EV standard from the urine of healthy donors with a primary particle concentration of 1.4 × 10^12^ particles mL^−1^ was purchased from Galen Molecular (North Haven, CT, USA). Mesh formvar carbon-coated copper grids and solvents used for TEM, which included 2% paraformaldehyde in PBS (16% stock) and 1% uranyl acetate (powder) in PBS, were purchased from Electron Microscopy Sciences (Hatfield, PA).

### Instrumentation

A VWR symphony 4417 tabletop centrifuge (Radnor, PA, USA) was used for the skimming of bovine milk and pelletizing of casein precipitants. Chromatographic separations were performed using a Dionex Ultimate 3000 HPLC system including a LPG-3400SD quaternary pump and MWD-3000 UV–Vis absorbance detector (Thermo Fisher Scientific, Sunnyvale, CA, USA) controlled by Chromeleon 7 software. For Bradford purity assays, an Agilent BioTek Synergy LX Multi-Mode Reader (Santa Clara, CA, USA) at 595 nm was employed. The Nanoflow analyzer (NanoFCM, Inc. Nottingham, Nottinghamshire, UK) provided information regarding size distribution, particle count, and immunodetection. Finally, a Hitachi HT7830 transmission electron microscope (TEM) (Chiyoda City, Tokyo, Japan) confirmed the size and structural integrity of individual MDEVs.

### Polyester C-CP fiber columns

The same types of C-CP fiber columns employed previously in exosome isolation protocols were employed in this effort [19, 20]. The method of column preparation has been described in detail previously [23]. Polyether(ether) ketone (PEEK) microbore HPLC columns are 30 cm in length and have an inner diameter of 0.76 mm. The eight-pronged shape of the hydrophobic, melt extruded polymer fibers allows for the interdigitation of individual fibers, increasing column surface area and solute/surface interactions by creating microchannels where the MDEVs can interact with the stationary phase [24].

### Bovine milk

MDEVs were isolated from raw bovine milk which was obtained through a collaboration with Farm Manager Reta Miller of the Clemson University’s LaMaster Dairy Piedmont Research and Education Center. Milk was collected in batches from Holstein cows via a Delaval V300 milking station (Tumba, Sweden) and pooled in a metal milk jug; 1 L of this milk was transferred to a 1 L glass bottle and transported 5 min by vehicle to the laboratory. Upon arrival to the laboratory, raw milk was aliquoted into 15 mL sample conical tubes, and the majority of the samples were frozen at − 20 °C for short-term storage while 5 were stored at 5 °C for immediate processing and analysis.

### Preparation of skimmed, raw bovine milk

As a first step in the reduction to the total fat content of the raw bovine milk, the fresh milk was skimmed twice using a tabletop centrifuge for 10 min at 1000 × g. The top layer of fat was carefully skimmed off after each centrifugation step using a spatula. The skimmed milk was then syringe filtered using 0.22-µm PES filters and diluted 1:1 with 1X phosphate-buffered saline (PBS, pH 7.4) for further processing. Heretofore, this material is referred to as filtered, skimmed, and diluted milk (FSM).

### Acetic acid precipitation of prepared milk

**Fig. 1:**
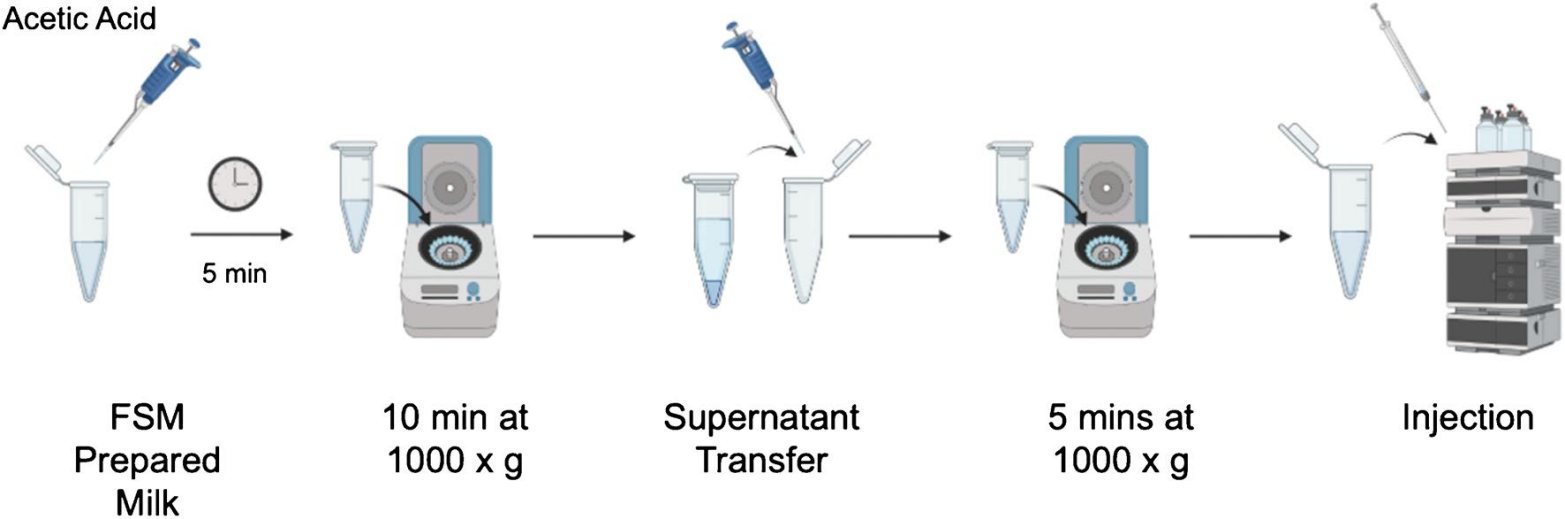
Depiction of the ultimate sample processing of bovine milk for the C-CP fiber procedure. One hundred microliters of the supernatant was injected on to the polyester C-CP fiber column mounted on an Ultimate 3000 HPLC for isolation

As noted above, the use of acidic precipitation has been demonstrated as an effective procedure to affect the reduction of the casein-based micelle content [17]. The basic processing steps in preparation for on-column injection are depicted in Fig. 1. In preparation for acetic acid (Ac) precipitation, 940 µL of FSM was aliquoted into a biocompatible polymer microcentrifuge tube. Glacial acetic acid was added for protein precipitation in (6% v/v) to the FSM and incubated on the benchtop for 5 min. Precipitated milk was then centrifuged for 10 min at 1000 × g to pelletize the protein precipitant. Following this first centrifugation step, the supernatant was transferred to a new microcentrifuge tube and the precipitant was discarded. A second, polishing centrifugation step was performed for 5 min at 1000 × g before injection onto the HPLC.

### HIC liquid chromatography method

The HIC separation method used in this investigation was developed based on previous work for the isolation of exosomes from human blood serum and plasma [18]. The ultimate chromatographic method is as follows. First, the column is equilibrated at a flow rate of 0.5 mL min⁻^1^ in 2 M ammonium sulfate (AMS) in 1X PBS which serves as the starting mobile phase. The 10-min step-gradient method begins upon the injection of 100 µL of sample into the high ionic strength carrier of 2 M AMS, which is held for 1 min. The protein elution step is a 4-min, lower ionic strength step with the addition of a mild organic modifier (1 M AMS and 20% ACN in 1X PBS). A 1-min PBS wash step is included between the 5 and 6-min points to avoid the precipitation of the AMS mobile phase with the increased levels of ACN content. Exosome elution consists of a 4-min step made up of 40% ACN in 1X PBS, bringing the total method time to 10 min. Exosomes were detected post-column through their optical absorbance (actually scattering) measured at 216 nm [19].

### Validation of vesicular structure

Transmission electron microscopy was used to verify the physical characteristics of recovered EVs by negative staining as detailed in Jung et al. [25]. Low-resolution images of raw milk products were obtained at the Clemson Advanced Materials Research Laboratory, where carbon-coated copper grids were incubated with 5 µL of sample for 20 min before blotting dry; a second aliquot was then incubated on the grid for 10 min and once again blotted dry. The grids were then washed with DI water and flipped upside down to rest on top of a drop of 2% paraformaldehyde for 5 min. Next, the sample was washed three times with sterile PBS and then rinsed with three drops of water, letting the grid sit in the last drop upside down for 2 min. A 0.22-µm-filtered 1% uranyl acetate negative staining solution was pipetted on the grid and allowed to rest for 15 s before blotting and drying. Sample grids were stored in the dehumidifier overnight and imaged the next day via TEM (HT7830) at 120 keV.

High-resolution TEM imaging of EV isolates was performed at the University of Washington Fred Hutch Cancer Center (Seattle, WA). Samples were prepared for imaging as previously described [26], with several modifications. Here, 30 µL of sample solution was deposited onto formvar/carbon-coated copper grids (Ted Pella, Redding, CA) for 30 min at room temperature. Samples were then rinsed three times with filtered PBS and fixed on drops of Karnovsky’s fixative for 5 min. Eight rinsing steps were done using drops of distilled water. Samples were then contrasted using 2% uranyl acetate/0.075 M oxalic acid at pH 7 through incubation for 5 min in 50 µL drops. Grids were then embedded by placing them on 50 uL drops of 0.4% uranyl acetate in 2% methyl cellulose on ice for 10 min. Grids were removed with wire loops, drawn on the edge of filter paper to remove excess liquid, and allowed to air dry. Samples were then examined on a Thermofischer Talos L120c transmission electron microscope (Thermo Fisher Scientific, Waltham, MA) at an accelerating voltage of 120 kV. Digital images were acquired with a Ceta 16 M CMOS 4kx4k digital camera system.

### Size distribution, particle count, and immunoconfirmation

Size distribution, particle count, and immunoconfirmation of the exosome isolates were determined using the NanoFCM Nanoanalyzer. The NanoFCM is a nanoflow cytometer with several bandpass filters that can simultaneously detect side scattering (bandpass filter, 488/10 nm), green fluorescence (bandpass filter, 525/40 nm), and red fluorescence (bandpass filter, 670/30 nm). The instrument is calibrated before each use with 1:100 dilutions in DI water of 0.25 µm fluorescent silica microspheres (QC Beads) and silica nanosphere cocktail (S23M-sEV) sizing beads. All solvents were filtered using a 0.22-µm PTFE hydrophilic syringe filter. The presence of vesicular bodies was confirmed using MEMGlow™ dye, while exosome identity was confirmed using an anti-CD81 fluorescent antibody. MEMGlow™ dye was used in a 1:2500 final dilution to dye the vesicle membranes and was analyzed in the 640–670 nm PE-Cy5 channel at 630 nm excitation and 680 nm emission. CD81 on the surface of the vesicles was detected using anti-CD81 ExoBrite fluorescent flow antibodies (Ab) in a 1:5000 final dilution using the 525–540 nm FITC channel at 490 nm excitation and 516 nm emission. Exosome fractions diluted to ~ 2.0 × 10^8^ particles mL⁻^1^ from HIC separations, which had 40% ACN evaporated off overnight, and were protected from light, and incubated at 37 °C for 2 h after labeling with anti-CD81 Ab and MEMGlow™. Samples were then gently vortexed and introduced to the flow cytometer according to manufacturer’s instructions. Operation of the instrument and data analysis was controlled via the NF Profession 2.3 software. Instrumentation blanks were acquired via injection of neat, 1X PBS solutions and EV isolates which were not fluorescently tagged.

### Particle quantification via method of standard addition

The total particle concentration in EV fractions was also quantified in-line using absorbance detection at 216 nm [19] and employing the method of standard addition. Three samples were made which contained 10 µL of 6% Ac pre-treated milk. Each sample included an exosome standard spike with a concentration of 1.4 × 10^11^ particles mL^−1^ (exosome spikes were 10 µL, 20 µL, and 30 µL respectively). All samples were then diluted to 100 µL using 0.22 PES filtered 1X PBS. Samples were then injected into a 20 µL loop and onto a new PET C-CP fiber column that was previously equilibrated in 2 M AMS for HIC isolation. A response curve was then constructed, and the data was extrapolated to determine the particle concentration of MDEV fractions.

### Protein content

The protein content in the milk and the exosome isolate fractions were quantified using a Bradford assay. During HPLC separation, fractions from the unretained injection effluent, the protein elution, and the exosome elution steps were collected by hand to measure protein content. The 20% and 40% acetonitrile from both the protein and EV elution fractions was evaporated off overnight at 5 °C, leaving vesicles in a 1X PBS solution. A β-casein response curve from 25 µg mL^−1^ to 2 mg mL^−1^ was generated according to the Thermo Fisher Scientific Coomassie (Bradford) Protein Assay Kit protocol. Five microliters of each standard and sample were added to the 96-well plate in triplicate. Following standard and sample loading, 250 µL of Pierce™ Coomassie Plus Bradford Assay Reagent was added to the wells and incubated at room temperature for 10 min, after 30 s of orbital shaking at 282 cpm. Once the 10-min incubation period elapsed, the optical absorbance was measured at 595 nm (*n* = 3) using the Agilent BioTek Synergy LX Multi-Mode Reader (Santa Clara, CA, USA).

### Determination of casein content

Immunoblotting was employed to assess the presence of casein within the MDEV isolates. Briefly, RIPA lysis buffer was added to lyse the MDEV eluates and a microBCA kit (Thermo Fisher Scientific, Waltham, MA, USA) utilized to measure the total protein concentration within the MDEV lysate. From these measurements, the total protein within the MDEV isolate fraction was determined. Based on that value, an aliquot equivalent to 500 ng of total EV-related protein and 500 ng of resuspended, purified casein protein were separated via SDS-PAGE. The separated proteins were transferred onto a PVDF membrane and incubated in blocking buffer consisting of 5% bovine serum albumin diluted in Tris-buffered saline with Tween 20 (TBST). After membrane blocking, casein was identified using a rabbit polyclonal anti-casein primary antibody (1:2000 dilution, ab166596; Abcam, Cambridge, UK), followed by incubating the membrane with horseradish peroxidase (HRP)–conjugated goat anti-rabbit IgG secondary antibody (1:10,000 dilution, HAF008; Novus Biologicals, Littleton, CO, USA). An ECL kit (Immobilon ECL UltraWestern HRP Substrate; MilliporeSigma, Billerica, MA, USA) was utilized for detecting HRP and a ChemiDoc system (Analytik Jena US, Upland, CA, USA) used for imaging analyses. Densitometry was performed using NIH ImageJ software, with the ratio of casein protein detected from the MDEV isolate band compared to the band of casein protein generated from the 500 ng of purified casein to assess the final casein content.

## Results and discussion

The lack of a standardized, field-wide protocol regarding the study of exosomes, including isolation and characterization of the vesicles, introduces an added layer of difficulty in moving putative vectors in a direction where downstream application is practical. Proposed methodologies require exosomes that include high particle counts with appropriate size distributions and intact biophysical morphologies, retention of surface protein activity, and high purities. Here, a novel approach to rapidly isolating high-purity MDEVs that are amenable to downstream applications is described.

### Chromatographic method development

**Fig. 2:**
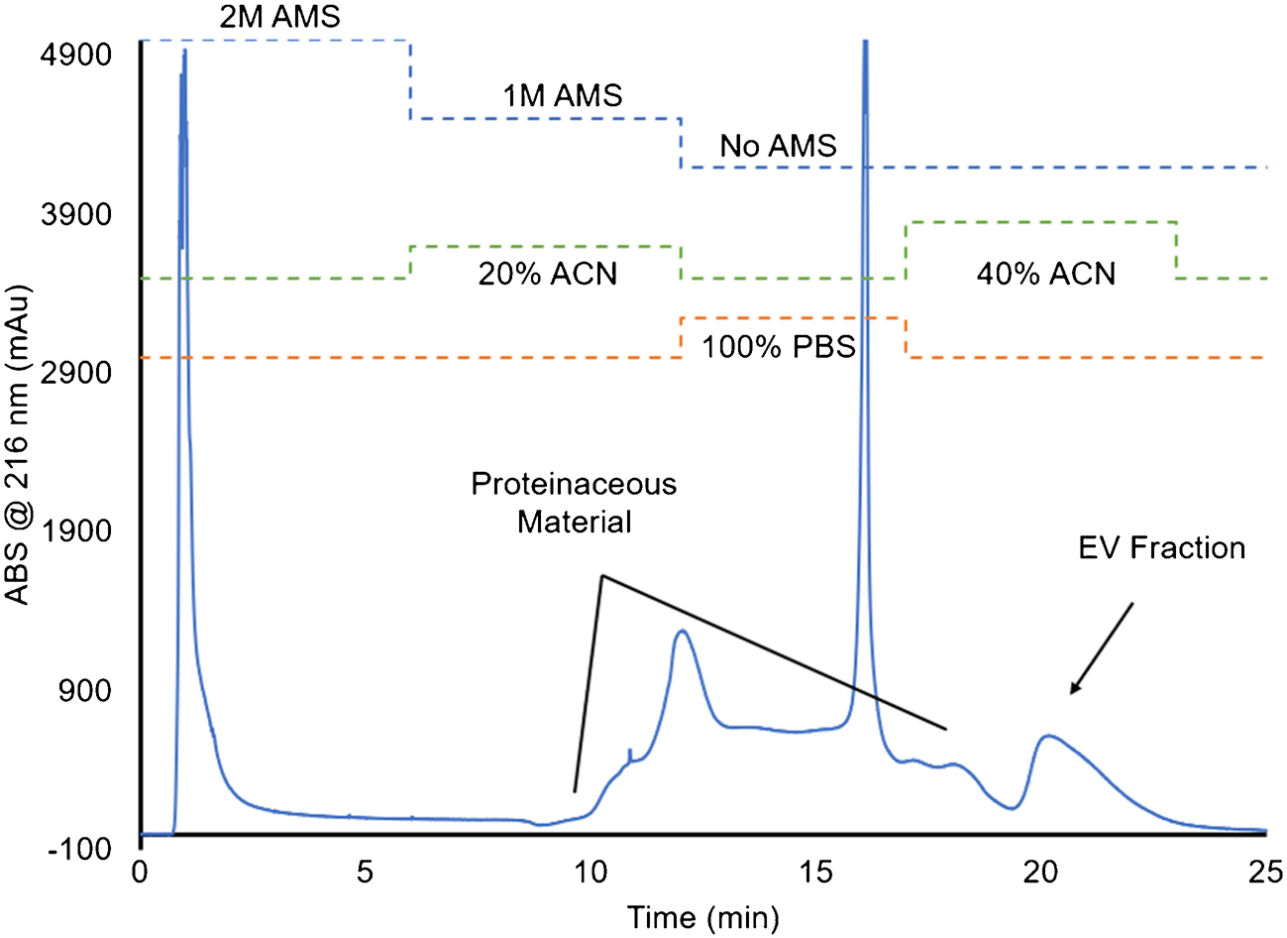
HIC chromatogram of raw, filtered skimmed milk for the isolation of milk-derived exosomes via PET C-CP fiber columns. Injection volume = 20 μL. The onset of species’ elution of ~ 3 min after onset of gradient steps reflects the transit time through the fluidics/column

The initial separation of exosomes from raw milk involved the HIC method developed by Bruce et al. wherein acetonitrile (ACN) was employed as the organic solvent modifier and the operations performed at a flow rate of 0.5 mL min⁻^1^ using the Dionex Ultimate 3000 analytical-scale liquid chromatography instrument [19]. A successful EV HIC separation produces three identifiable absorbance signal transients: an injection peak reflective of any unretained species, a protein elution band, and the exosome elution peak. One modification to the initial step-gradient method has been the addition of a short (1 min), 100% 1X PBS plug which serves to ensure better solvent mixing before the last gradient step. Ideally, one would wish to do the analyses without any modification/manipulation of the raw milk. Perhaps not surprisingly, this resulted in the immediate clogging of the column, even with an in-line 0.22-µm filter. Based on inferences in the literature, the raw bovine milk was skimmed by centrifuging twice for 10 min at 1000 × g and filtered using a 0.22-micron PES syringe filter before injection. Skimming of the milk completely alleviated column clogging and the result of the initial chromatographic method employed for the MDEV isolation on the C-CP fiber columns is presented in Fig. 2. The first signal on the chromatograph is indicative of unretained species (the injection peak), as would be expected. Very different from previous efforts including urine, plasma, and cell culture matrices, there is not a clear, singular protein elution band as expected when the concentration of ACN was increased at *t* = 6 min. However, there is a very intense, diffuse-in-time, absorbance band presumably due to the elution of matrix species. The fact that a pair of distinct peaks is seen would reflect the presence of discrete and diverse populations of chemical species that might include proteins, fats, and micelles. The final band eluting at ~ 20 min will be shown to be representative of the target exosomes, but given the peculiarities of the rest of this chromatogram this assignment would be quite speculative.

**Fig. 3:**
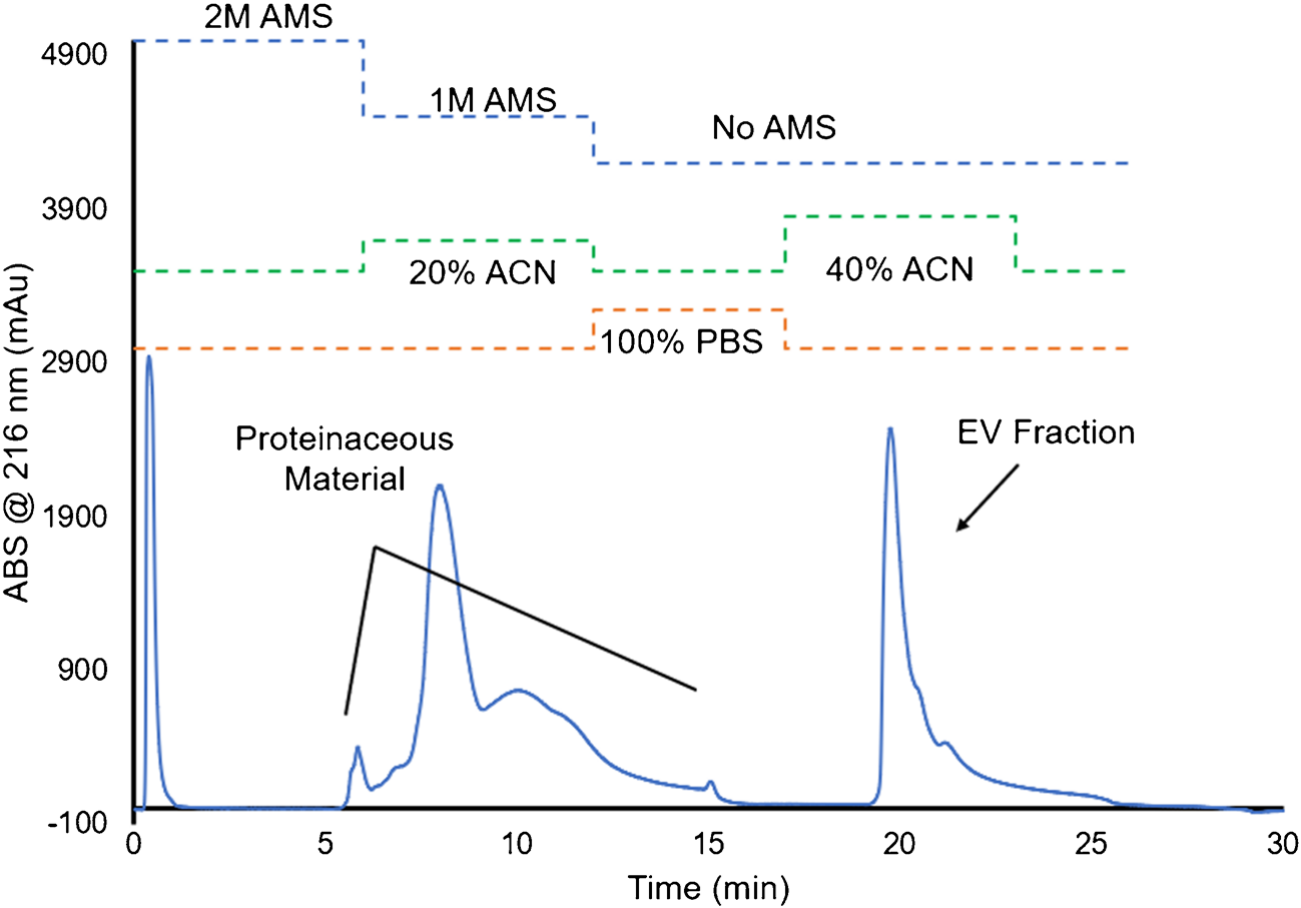
HIC chromatogram of 1:1 dilution of raw, filtered skimmed milk in 1X PBS for the isolation of milk-derived exosomes via PET C-CP fiber columns. Injection volume = 100 μL. The onset of species’ elution of ~ 3 min after gradient steps reflects the transit time through the column

Due to a large amount of matrix-species co-elution occurring in the isolation of the filtered, skimmed milk, that matrix was diluted 1:1 with 1X PBS. In this trial, the chromatographic profile presented in Fig. 3 is similar to the milk that was undiluted, but with better segregation between the protein and EV elution bands. The ability to inject a 100 µL volume versus the 20 µL of the undiluted sample (Fig. 2) reflects the much lower viscosity and solid content, and a greatly reduced propensity for column fouling without altering EV purity. Additionally, some of the effects of the milk’s heavy matrix were minimized and yielded a much more defined exosome elution peak, showing promise for the use of HIC separations to isolate MDEVs. When compared to EV isolates from other matrices like plasma, urine, and cell milieu, which yield anywhere from 5.0 × 10^10^–2 × 10^12^ particles mL^−1^, the bovine milk isolation yields far greater absorbance response [18–20]. That said, and as described earlier, casein micelles, which mimic physical properties of EVs, might co-elute with the desired vesicles [11, 12] impacting particle counts and vector purity suggesting the need for a more rigorous pre-treatment of the raw bovine milk to better ensure removal of residual casein.

**Fig. 4:**
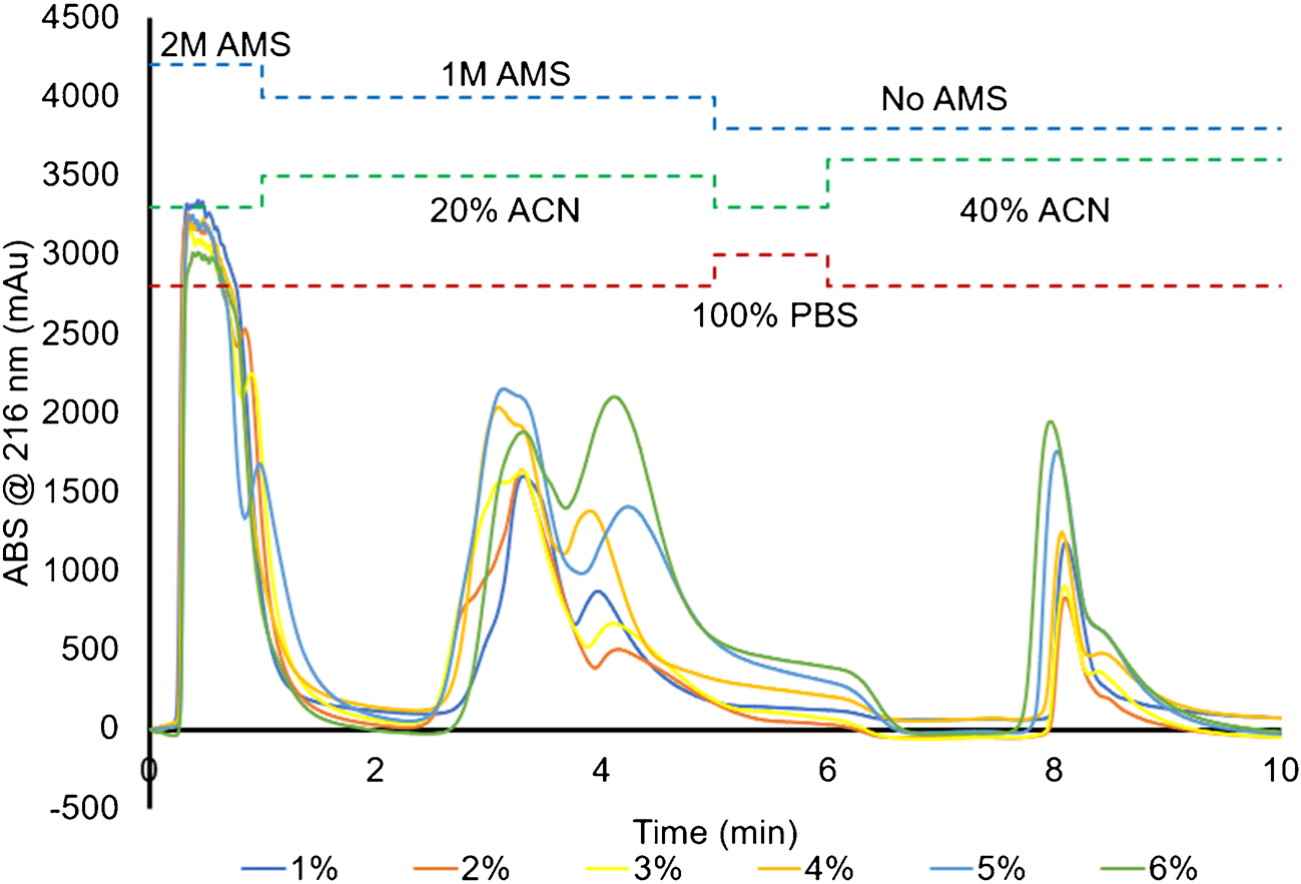
Representative HIC chromatograms of 1:1 1X PBS diluted raw, skimmed, and filtered milk treated with increasing concentrations of 1–6% acetic acid prior to injection. Injection volume = 100 μL

Removing additional casein micelles and extraneous protein species with acetic acid (Ac) precipitation, as described previously [12, 16], should improve matrix-related species removal to produce more effective vector isolation, while still being time and cost-efficient. By precipitating the casein micelles via Ac precipitation after skimming and filtering the raw milk, the purity and yield of isolated EVs are improved. In addition, the initial retention of those “impurities” on the fiber surfaces compromises the binding capacity for the target EVs. Concentrations of Ac from 1 to 6% v/v were added to the diluted, FSM samples to effect the precipitation of proteins, with representative HIC chromatograms performed on the PET C-CP fiber columns presented in Fig. 4. Here, the gradient used to isolate MDEVs was shortened significantly to maximize throughput. The injection and PBS steps were shortened to 1 min each, while the protein and EV elution steps were shortened to 4 min each, reducing the total separation time to 10 min. As expected, when the concentration of the added Ac was increased, the amount of nominally hydrophobic matrix species and proteinaceous material removed in protein elution steps increased. Specifically, the integrated area increased by a factor of ~ 2.4 × between the 1 and 6% Ac treatments. More impacted, and relevant, is the increase in the EV recovery (based on integrated absorbance) by a factor of 3.7 × at the highest Ac content. It must be mentioned that the values quoted are for the average of 3 injections at each Ac percentage level. The greater yield in MDEV recoveries based on the treatment with acetic acid may well be reflective of lesser quantities of proteinaceous material being retained on the fiber surfaces prior to its elution. Finally, it should be noted that even the lowest concentration of acetic acid resulted in a much reduced response for the supposed EV elution band (i.e., versus Figs. 2 and 3), which seems to point to the fact that the fraction was indeed likely also composed of casein-related micelles having similar hydrophobicities to the target EVs, and that these species were appreciably effected by the acid treatment as hypothesized.

**Fig. 5:**
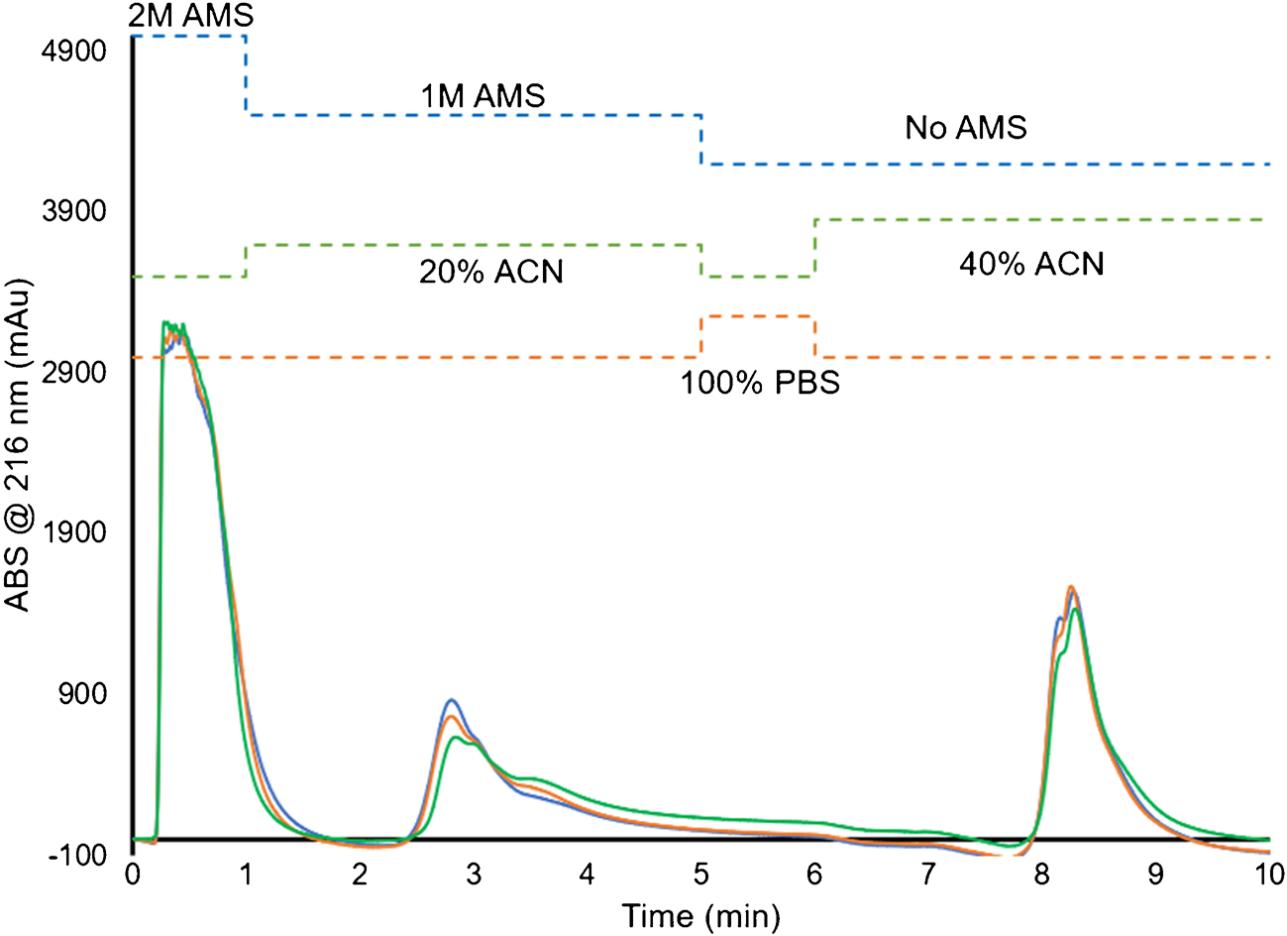
Representative HIC chromatograms for triplicate preparations of 1:1 1X PBS diluted raw, skimmed milk treated with 6% acetic acid, and filtered prior to injection. Injection volume = 100 μL

The complete, optimized sample preparation workflow is presented in Fig. 1 where 940 µL of FSM is pre-treated with 6% v/v acetic acid (60 µL) and incubated at room temperature for 5 min before being centrifuged for 10 min at 1000 × g. Following the centrifugation step, the supernatant was carefully removed from the microcentrifuge tube, avoiding the precipitant on the sidewalls. The supernatant was then transferred to a second microcentrifuge tube to pellet any remaining precipitant at 1000 × g for 5 min. The sample was taken directly from the second microcentrifuge tube for HPLC analysis. It is important to note that the sample preparation to this point is no more cumbersome than the other cited approaches, but the actual HIC separation provides very high improvements in process throughput. Figure 5 demonstrates the reproducibility of the 6% Ac pre-treatment method in triplicate extractions (i.e., the complete analytical process) and the HIC separations from the same bovine milk sample. In this process, the intrasample precision of multiple injections of the same preparation varied by less than 5%RSD, with the intersample variability across the three preparations varying by 9.7%RSD, here. Based on a detailed literature search, the relative precision for other analytical MDEV separation methods could not be found.

### Confirmation of vesicular structure

**Fig. 6:**
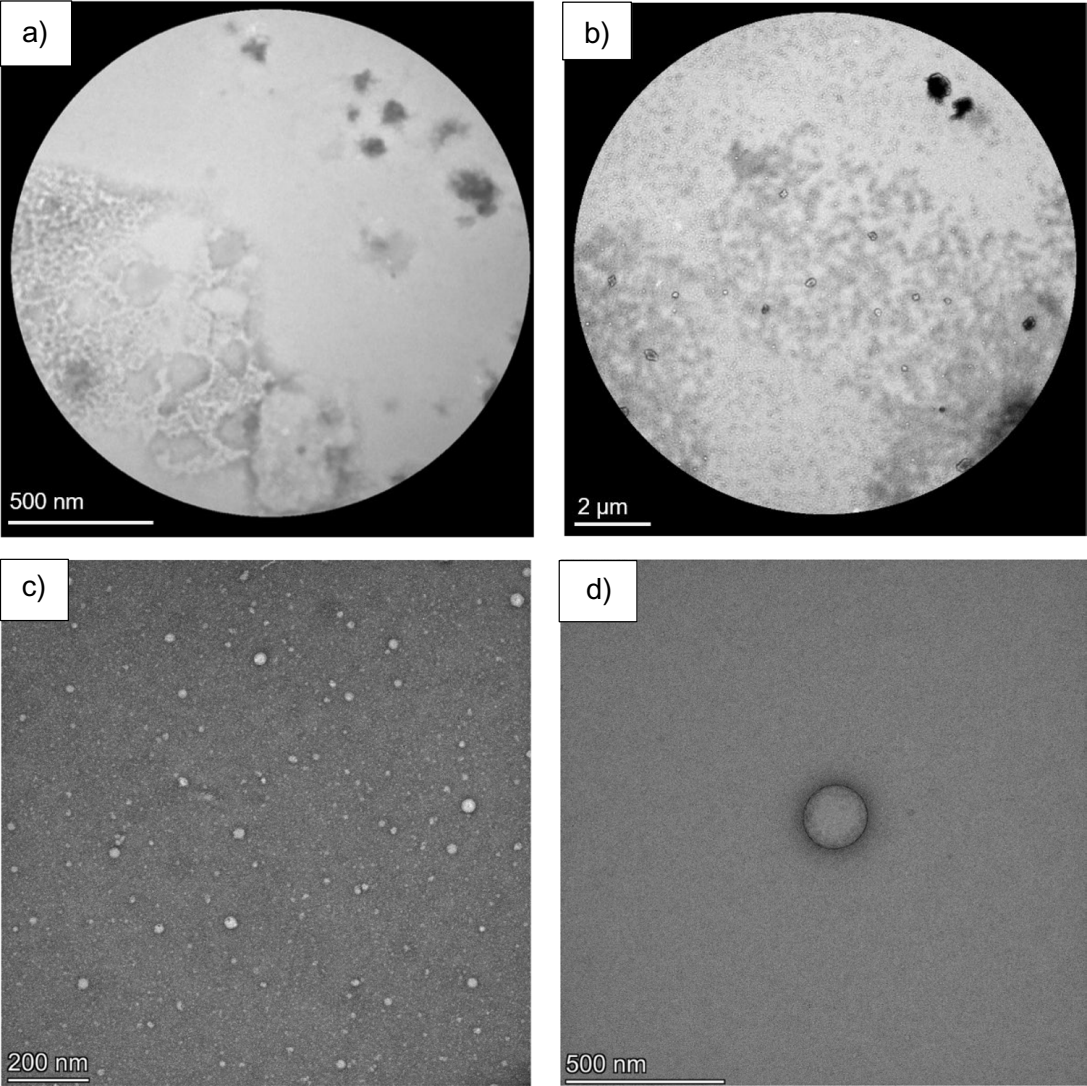
TEM images of **a** diluted raw milk before acid precipitation (500 nm scale), **b** image of diluted raw milk after acid precipitation (2 µm scale), **c** a diverse population of isolated EVs demonstrating a size range (30–200 nm) expected of exosome populations (200 nm scale), and **d** a single EV post-isolation which clearly depicts an intact lipid bilayer and cup-like shape (500 nm scale)

The first level of success and validation of any exosome isolation process is the recovery of intact vesicles. TEM imaging is effective in highlighting the vesicular structure of EVs, but also registers responses of what are otherwise undesirable particles, and is useful in illustrating the efficacy of the present exosome isolation strategy. Transmission electron micrographs of 1:1 1X PBS diluted raw milk and exosome isolates from the EV fraction were taken using the Hitachi HT7830 TEM (note differences in scale sizes among the micrographs in the caption). The micrograph of the raw milk (Fig. 6a) required prior dilution because the sample’s high viscosity impaired imaging after negative staining and caused many of the subsections within the grid to rip. On the left side of this image are what appear to be fat globules distributed next to large dark spots which are likely protein and/or large micelle aggregates [27]. After Ac pre-treatment, these fat features are not observed, and protein/micelle aggregates are minimal as seen in Fig. 6b. Figure 6c demonstrates the efficacy of the developed isolation method encompassing a wide population of representative MDEVs, which exhibit the size heterogeneity expected of exosomes, lying within the scale of 30–200 nm. These vesicles display lipid bilayer membranes that are intact, i.e., not ruptured, sheared, or “rough” as previous acetic acid precipitation methods have described [16]. A biophysical property of exosomes, a preserved “cup-like” shape, is also captured in these images. Figure 6d is an image of a single MDEV within the frame following the Ac pre-treatment and C-CP fiber tip HIC exosome isolation. Here the vesicular structure is clearly seen, indicating the efficacy and non-denaturing effects of the Ac pre-treatment and isolation methods.

### Particle characterization and immunoassay

The injection peak, protein elution, and MDEV elution fractions were collected for each of the samples separated by the 10-min method presented in Fig. 5, and subjected to the standard characterization slate in terms of confirmation of vesicular structure, particle sizing and immunoassay, particle count/concentration, and EV purity.

**Fig. 7:**
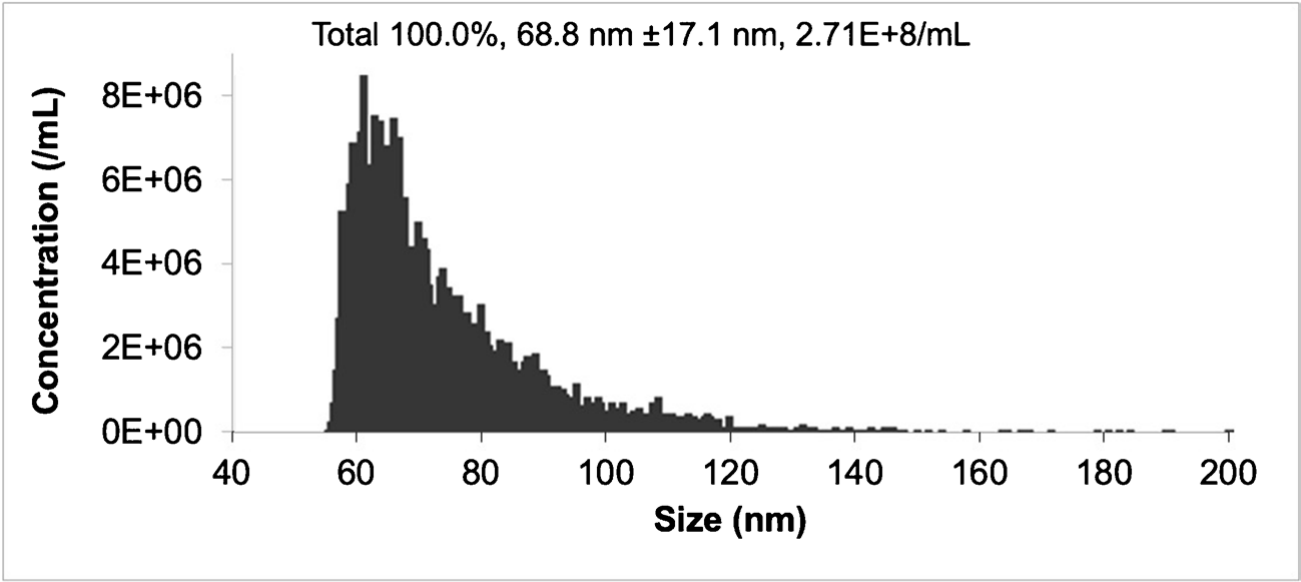
Histogram of particle size distribution measured via light scattering using the NanoFCM Nanoanalyzer, showing the median result from *n* = 3 trials. Calibration data indicate a particle density of 2.71 × 10^8^ particles mL^−1^ and an average particle diameter of 68.8 nm with a mode of 53.8 nm

The NanoFCM Nanoanalyzer instrument provides a great deal of complementary information in its ability to perform particle sizing and density determinations via light scattering, and identification of membrane-bound tetraspanin proteins via immunofluorescence. Figure 7 depicts one representative set of light scattering data (among the triplicate extraction/separations), where the system (following calibration) reports a histogram of the responses, particle sizing statistics, and the integrated particle density referenced to a 1 mL sample volume. Across the triplicate separations, the average particle count as determined by NanoFCM was 2.7 × 10^8^ particles mL^−1^ with an RSD of 9.5%, which directly coincides with the reproducibility observed in the integrated absorbance values across the triplicate samples. The absolute values reflect higher MDEV particle recoveries than reported for ultracentrifugation isolation (10^6^ mL^−1^) in the evaluation of the acetic acid pre-treatment procedures [22]. Recognizing that the NanoFCM analyzes EV eluates post-column that are diluted > 6 × over the course of the separation, the method of standard addition was utilized to further quantify the particle count of isolated, 6% Ac pre-treated MDEVs via absorbance. As an orthogonal method of quantification, samples of 1:10 diluted 6% Ac treated milk were spiked once, twice, and three times with exosome standard from the urine of healthy donors (1.4 × 10^11^ particles mL^−1^) to create the response curve. The response curve had an *R*^2^ value of 0.9815 and yielded a particle count of 1.5 × 10^10^ particles mL^−1^ for the triplicate MDEV samples. Quantification of particle concentration via NanoFCM is limited by the standards used to calibrate the instrument; the S23M-sEV standard has a size range of 53–120 nm, making it difficult to accurately determine the particle count of EVs (30–200 nm) outside the range of the standard. It is unclear whether vesicles smaller than the lower limit of the sizing standard are isolated due to instrument limitations. The limitation of detecting EVs in the standard’s size range is important to note, because the lower range (30–60 nm) of small EVs (30–150 nm) make up 10–15% of MDEVs present in milk, indicating size information is accessible for ~ 85% of vesicles detected [8]. The histogram shown here is the result of a single analysis that was replicated (*n* = 3) to ensure reproducibility. Absorbance quantification does not have the same calibration constraints as nanoflow cytometry; by spiking treated milk samples with EVs of known concentrations and then performing a separation to create a response curve based on EV elution absorbance values, it ensures that large populations of small EVs are not excluded from total yields.

The average particle diameter across the triplicate exosome fractions was 68.6 nm, with a variability of 0.41% RSD. The precision of both sets of measurements is considered excellent in comparison to reports of other methods like nanoparticle tracking analysis (NTA) [15, 28]. A size distribution in the range of 30–200 nm is on par with the expected size range of EVs from other vesicle sources, though a limitation of this measurement is the NanoFCM S23M-sEV size standard, where vesicles smaller than 53 nm cannot be accurately sized [16, 29].

**Fig. 8:**
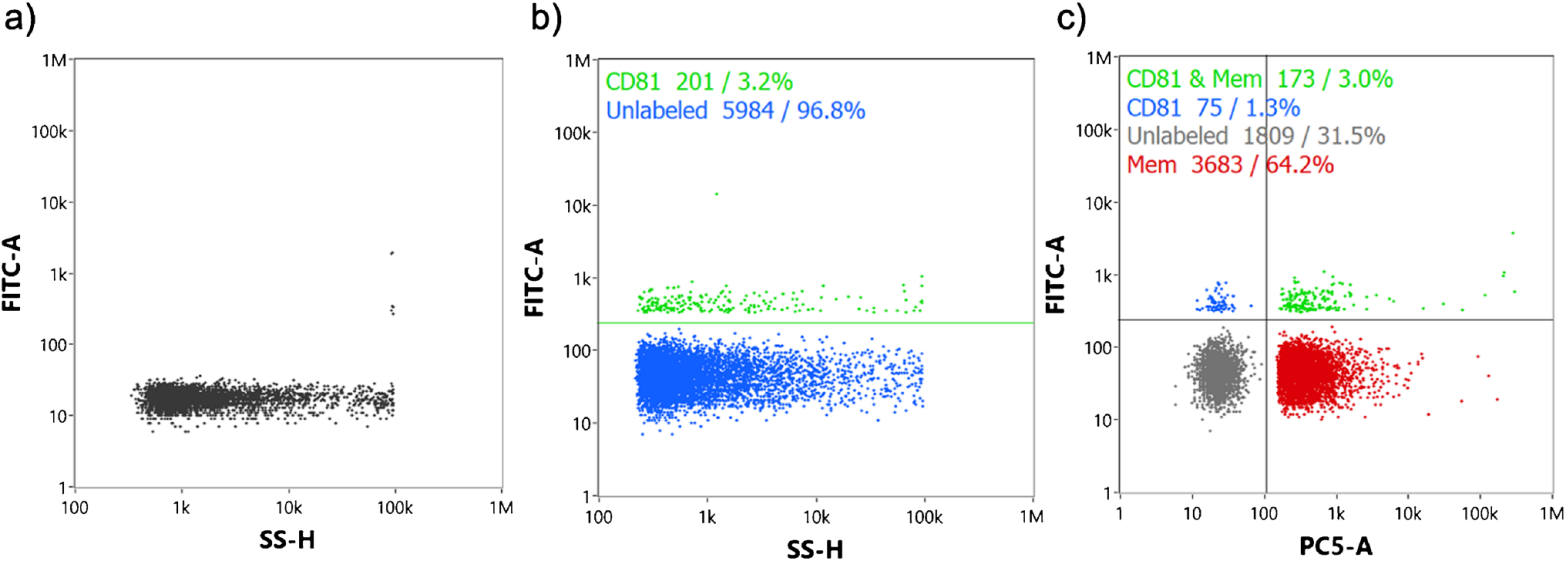
Representative NanoFCM-generated dot plots of fluorescence response of complete process isolates. **a** Measurement blank wherein no labeling is performed. **b** Identification of MDEVs based on the labeling with anti-CD81-FITC Ab. **c** Comprehensive identification by dual-labeling with MEMGlow™ and anti-CD81-FITC Ab

To confirm that the particles measured were indeed exosomes, they were subjected to immunofluorescence assays using a membrane dye to verify vesicular structure and fluorescently labeled antibodies for the known exosome tetraspanin CD81; effectively a nanoflow cytometry determination. Immunodetection via the NanoFCM positively identified the vesicles via the MEMGlow™ membrane dye and anti-CD81-FITC Ab tags. The experimental controls implemented included samples blank in both anti-CD81 and MEMGlow™. Figure 8a is a dot plot of an exosome negative control, where the EVs were not incubated with either MEMGlow™ or anti-CD81-FITC Ab before NanoFCM analysis. As expected, there is a 99.8% response for FITC (-) particles, with aggregates making up the remaining 0.2% of the population. This, in and of itself, provides support for the efficacy of the MDEV isolation process. For a first level of confirmation, the captured MDEVs were incubated with the anti-CD81 fluorescent tag. Figure 8b demonstrates a 3.2% positive anti-CD81-FITC Ab response, a value which is in line with what would be expected for this set of incubation conditions and the previously noted Ac precipitation work. More definitive of the identity of the isolates, immunodetection of bovine MDEVs using the two different biological probes, was performed, with the resultant scatter plot presented in Fig. 8c. A positive response for MEMGlow™ was observed in 64.2% of the sample population. MEMGlow™ membrane dye and anti-CD81-FITC Ab both show a correlated, positive response of 3.0%, indicating that the population of exosomes were in fact vesicles with phospholipid bilayers, and contained biomarkers native to EVs. In this regard, the correlation is clearly limited by the efficacy of the antibody labeling step. These results indicate that isolates from the pre-treated bovine milk contained an exosome subpopulation which retained its biological identity/activity post-isolation as indicated by positive fluorescent responses, which would not be observed if the Ac pre-treatment had denatured membrane protein markers. To be sure, the use of multiple immunofluorescent labels would provide greater levels of certainty in the identification of specific EV populations, and this will be applied in future efforts. Ultimately, when considering the application of exosomes as putative therapeutic vectors, it is imperative that the EVs retain their biological activity, as surface protein and ligand interactions facilitate their movement through cellular communication pathways [1]. Demonstrating that the acetic acid pre-treated and acetonitrile isolated exosomes do indeed retain their ability to bind with Ab and uptake the membrane dye suggests promise for their successful reintegration into cellular communication pathways; of course, this fact must be confirmed in detail.

### Determination of recovered EV purity

To determine the purity of the isolated vesicles and the efficacy towards casein removal, Bradford assays were performed, with the fate of the total protein content mapped across the entire purification process; raw milk, FSM treated with 6% acetic acid, the protein fraction eluted from the column, and the final collected EV fraction and its RIPA lysate. It is important to note that a positive response from the Bradford assay is anticipated in all fractions, as the Coomassie reagent responds to “free” proteins in solution as well as produces a positive response towards the proteins embedded in the exosome vesicular membranes (e.g., tetraspanins). On the other hand, the protein content of the lysate reflects the free proteins and the internal contents of the vesicles as well as the membrane-bound proteins. In preparation for the Bradford assay, fractions that contained acetonitrile (i.e., the protein and EV elution fractions) were stored in an open vial at 5 °C, overnight, to allow the solvent to evaporate.

**Fig. 9:**
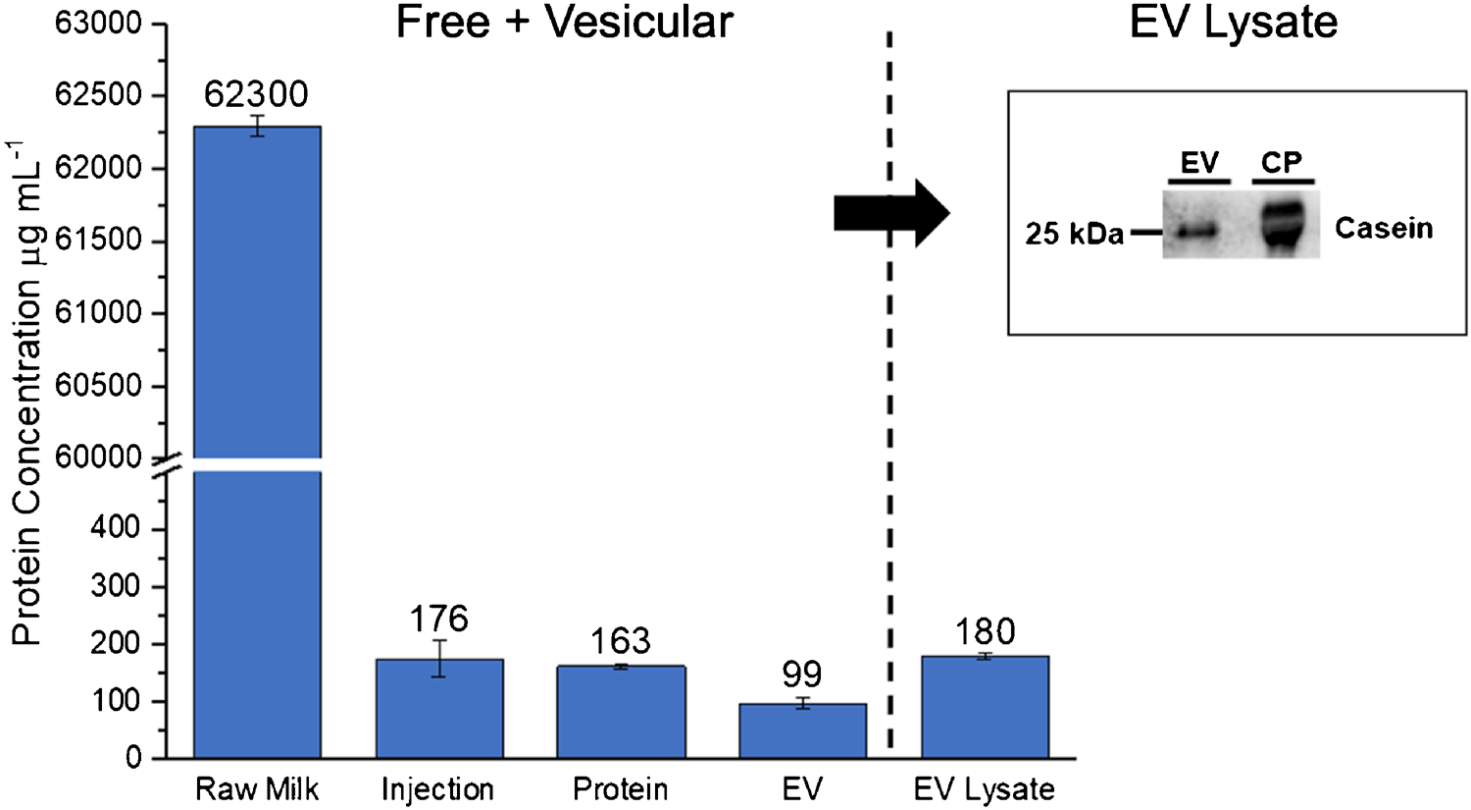
Total protein content in µg mL^−1^ via Bradford assay of raw milk, as well as the 6% Ac pre-treated milk injection, protein, and EV fractions from PET C-CP fiber column isolations. The micro-BCA assay results from the RIPA-lysed EV isolates are depicted as well as a representative, comparative SDS-PAGE/immunoblotting image for 500 ng loadings of the EV lysate and pure β-casein. Both protein assays used β-casein for the standard curves

The results of the Bradford assays for the processed fractions (averaged across the triplicate samples) are presented in Fig. 9. Here it is very important to reiterate that casein itself makes up approximately 80% of the initial protein content in bovine milk [11, 12]. Although the efficacy of the 6% precipitation is apparent in the dramatic reduction in the absorbance of the protein band in the chromatogram, the Bradford assay results provide further confirmation of the successful protein removal than the chromatographs suggest. It should be remembered that the Bradford assay itself is responsive to *amino acid* content, with the ultimate “protein content” value derived from calibration functions derived from serial dilutions of bovine serum albumin (BSA). However, considering casein is the target of the acetic acid precipitation pre-treatment, the serial dilutions for calibration were performed using β-casein from bovine milk. Before the comparative analysis, it must be emphasized that for each set of samplings/determinations, the standard deviations (denoted by error bars) in every case are less than 5% RSD. This reflects the excellent repeatability of the protocol developed here.

The results of the Bradford assay of the respective fractions are relatively straightforward and reflective of the overall process. As would be expected, the protein content of raw, whole milk is very high, with a baseline value of ~ 62,000 mg mL^−1^. Though this protein total may seem exaggerated, Bradford assays reflect the free amino acids, small peptides, proteinaceous, and amino acid rich somatic cell debris present in the milk [30]. Data collected from the LaMaster Dairy farm Holstein herd provided by the Lancaster Dairy Herd Improvement Association (DHIA) Lab reported that the herd is producing milk with fat content averaging ~ 4% and proteins accounting for ~ 3.2% of milk content, indicating that at least half of the protein content reported here, determined by the Bradford assay, are due to intact proteins identifiable by a Bentley FTS/FCM instrument which employs flow cytometry and Fourier transform spectrometry for analysis. The non-retained (post-column), 6% Ac-treated, FSM injection fractions showed a 350-fold reduction in total protein content versus the raw milk. While it would be expected that any remnant proteins would have been retained on the C-CP fiber column, these non-retained species are likely proteins broken down to simple peptide/amino acid forms, which are not likely retained on column but would still respond positively in the Bradford assay. The first gradient step affects the elution of intact, hydrophobic proteins, in this case yielding an average protein content of 163 µg mL^−1^. The ultimate, target exosome fraction responded to the Bradford assay to yield an average protein content of 99 µg mL^−1^ with respect to intact EVs (not RIPA-lysed).

Very clearly, the cumulative processes of filtering, skimming, and acetic acid precipitation are highly effective in reducing the free protein content in the bovine milk (> 99%), while also providing for very clean chromatographic separation of exosomes. Based on the EV particle densities determined via absorbance detection, a purity of 1.76 × 10^10^ vesicles per microgram of protein is calculated here. While such numbers are very difficult to find in the literature, the MDEVs isolated via PET C-CP fiber columns were comparable in purity to MDEVs which were concentrated post-isolation via SEC [31]. Previous work isolating exosomes utilizing PET C-CP fiber columns has seen the exosome fractions yielding lower remnant protein concentrations than the values reported here. This is not a surprise as the presence of greater amounts of transmembrane proteins is expected in milk EVs than in matrices like urine [11, 12]. Milk vesicles are enriched with surface proteins that facilitate vesicle fusion and transport; in fact, more than 2000 unique protein species have been identified on the surface of bovine milk–derived exosomes [32]. Therefore, it is not unexpected that the exosome fraction produced a greater positive response in the Bradford assay than the isolations of exosomes from other matrices, thereby influencing the purity of final exosome yields [18, 31].

The figures of merit with regards to the EV recoveries (~ 1.5 × 10^10^ mL^−1^) and purities (~ 2 × 10^10^ vesicles per microgram protein) are very respectable for such a simple and rapid separation process; the question of relevance in MDEV isolation methods reduces down to the extent of casein reduction. Having already demonstrated through nanoflow cytometry that EVs are indeed present; the goal of the immunoblot was casein identification and quantification in fractions of RIPA-lysed EVs (EV lysate). As seen in Fig. 9, micro-BCA assays of the lysates produced an average value of 180 µg mL^−1^ of total proteins/amino acids. This level of increase over the intact EVs is not surprising as proteins and amino acids make up an appreciate fraction of the vesicle content. In an attempt to quantify the relative amount of casein in the EV lysate, equal masses of protein of the lysate and pure casein were applied to the separation gel. Specifically, an EV-lysate volume equivalent to 500 ng of total protein and 500 ng of pure casein were analyzed. As seen in the inserted image, the amount of actual casein in the MDEV isolate is much less than the applied pure casein, with densitometer readings suggest values of ~ 17–20% relative to the applied target protein, i.e., ~ 100 ng of casein in the 500 ng of lysate protein. This result indicates that ~ 80% of the total casein present in the initial sample supernatant was removed via precipitation. It must be emphasized that while this result does indicate the presence of casein in our MDEV isolates, this finding does not determine the proportion of casein protein that exists as cargo within the membrane/cavity of the MDEV isolates versus remnant extravesicular (solution phase) casein protein. It is not outside of the realm of reality that casein would be a natural component of the internalized MDEV cargo. Indeed, this is a very challenging question throughout the field of EV proteomics and noted as well in MDEV proteomics experiments [33, 34].

## Conclusions

The interest in using milk-derived extracellular vesicles (MDEVs) as vectors in the gene therapy arena is increasing at an incredible rate, with primary drivers being the near-infinite, sustainable sourcing and natural immunogenicity of the exosomes [10, 35]. A major hurdle in the basic research regarding MDEVs, much less in their mass production for therapeutics, is isolating the vesicles from a matrix that is abundant in fats, proteins, and other matrix-related species [11]. High-fat content and colloidal structures in milk formed by casein micelles have made milk an exceptionally difficult matrix to tackle when it comes to EV isolation. Methods like UC, UF, SEC, and TFF have seen success in the isolation of EVs from diverse biofluids and cell culture media but experience challenges in producing the same product purity when confronted with the isolation of MDEVs. Often clinicians cannot wait 48 + h for the target vesicles to be isolated, or do not have the resources to use a new column for replicate separations from identical sources. Efforts described here hold promise in both areas; basic biochemistry and biotherapeutic developments.

Presented here is a novel method of MDEV isolation based on the performance of hydrophobic interaction chromatography on PET C-CP fiber columns mounted on a standard HPLC instrument. The pre-treatment of bovine milk with 6% v/v acetic acid followed by column purification yields exosome concentrations on the order of 1.5 × 10^10^ particles mL^−1^, a significant improvement over methods such as ultracentrifugation [22], with average diameters of ~ 68 nm. Their intact vesicle membranes and cup-like shapes observable by TEM further demonstrate the preserved biophysical characteristics after isolation. These exosomes contain the essential structural elements towards their biological activity, producing positive responses for exosome specific CD81 membrane proteins and MEMGlow™ vesicle membrane dye. Isolation of MDEVs was achieved in 10 min, with the use of only 5 mL of elution solvent. Reduced isolation time and solvent usage as well as the in-line column regeneration capability is a considerable advantage of using this method to isolate high-purity exosomes. The columns employed in these studies were employed for more than 15 cycles, without appreciable degradation in performance. Extended isolation times required by methods like ultracentrifugation can be a deciding factor in exosome applications when in reference to clinical settings [22]. By implementing reusable PET C-CP fiber columns, laboratories can increase output while driving costs down.

This initial effort in the use of C-CP materials for the isolation of MDEVs provides solid support for further investigations. Future efforts in the isolation of MDEVs via the C-CP fiber column platforms will focus on the clearly required affirmation that the isolated EVs are indeed viable for cellular uptake. Direct comparisons with other MDEV isolation methods regarding practical metrics, as well as potential biological aspects, are also warranted. Once the expected promise is confirmed, efforts will turn to scale-up of the columns for preparative applications. While the columns employed here have the binding capacity to isolate 10^12^ EVs from mL volumes, surely the processing of bulk milk products will require the processing of higher volumes that will necessitate greater column binding capacities. The physical structure of the C-CP fiber columns, having high permittivity to allow for lower processing backpressures and very low material costs, bode well for this avenue of development. These efforts are currently underway as recent studies have confirmed that basic hydrodynamic scaling laws (e.g., Darcy’s law [36, 37]) are obeyed regarding the packing of columns of different interstitial fractions and fiber types [38]. Success will ultimately depend on developing methods to ensure parallel packing of the fibers along the column axes for diameters upwards of 25 mm to achieve column loadings of > 10^15^ EVs and beyond.

## Data Availability

Data employed in support of this manuscript are housed in the research laboratory, and may be obtained by written request.
